# Development of a discharge education program using the teach-back method for heart failure patients

**DOI:** 10.1186/s12912-021-00622-2

**Published:** 2021-06-24

**Authors:** Eui Geum Oh, Hyun Joo Lee, You Lee Yang, Sewon Lee, Young Man Kim

**Affiliations:** 1 Mo-Im Kim Nursing Research Institute, Yonsei University College of Nursing, Seoul, Republic of Korea; 2 Yonsei Evidence-Based Nursing Centre of Korea: A Joanna Briggs Institute Affiliated Group, Seoul, Republic of Korea; 3 Department of Nursing, Seojeong University, Yangju, Republic of Korea; 4 College of Nursing, Eulji University, Seongnam, Republic of Korea; 5 Department of Nursing, Graduate School, Yonsei University, Seoul, Republic of Korea; 6 College of Nursing, Research Institute of Nursing Science, Jeonbuk National University, 567 Baekje-daero, Deokjin-gu, Jeonju-si, Jeollabuk-do 54896 Republic of Korea

**Keywords:** Heart failure, Program development, Self-management, Teach-back communication

## Abstract

**Background:**

Heart failure (HF) patients have difficulties in self-management after discharge. This study aimed to develop a discharge education program for HF patients using the teach-back method (TBM).

**Methods:**

As a methodological study to develop a program, we applied the analysis, design, development, implementation, and evaluation (ADDIE) model comprised of (1) analysis using EMR data, systematic review, and focus group interviews, (2) design and development of a program draft, (3) tests of program validity using 15 experts, 10 nurses, and 10 patients, and (4) development of the final program. The content validity index (CVI), and understandability and actionability of the educational material were used.

**Results:**

The discharge education program provides definitions and information about medication, symptom/weight/diet management, physical activity, and other precautions. The educational method uses TBM. The overall CVI for the program was 0.96, and all item CVIs were greater than 0.8. The understandability and actionability were 90.2 and 91.3 % in patients, and 94.6 and 86.8 % in nurses. The contents and methods of the program were appropriate for patients and providers.

**Conclusions:**

We expect the discharge education program using TBM to enhance self-management among HF patients. The process we used to develop this program could guide researchers and clinical practice.

## Background

Heart failure (HF) is a complex clinical syndrome resulting from structural and functional heart problems, such as impairment of ejection fraction in ventricles and a low level of tissue or organ perfusion [1]. The global burden of HF has been increasing, with 26 million patients suffering from HF globally [2]. Recently, governments and policymakers have begun to pay attention to the readmission rate of HF patients to reduce unnecessary health care costs [3]. The 30-day readmission rates for HF in developed regions, such as the United States (US) and Europe, are greater than 20 % [4]. In Korea, the prevalence of HF has been increasing steadily since 2002 [5], and the readmission rate of HF patients (27.6 %) is as high as in developed countries [6]. The US started the hospital readmission reduction program (HRRP), which reduces the payment to hospitals with excess readmissions, in 2012 and chose HF as a target disease. The Korean government has also used readmission rate as a hospital quality indicator since 2016. After the HRRP was implemented, the readmission rate of HF patients in the US decreased slightly, but their short-term mortality rate increased [7]. In other words, either hospitals did not allow hospitalization of severe HF patients, or high-quality care services are required to enhance self-care in the community and at home after discharge.

It is generally known that HF is a common disease in elderly patients [8], and that HF patients have difficulties in self-management because of the complexity of the disease characteristics [9]. Care for HF patients includes complex medication adherence, symptom management, weight management, dietary management, and physical activity [10]. Effective self-management interventions for HF patients could theoretically improve their health outcomes, including readmission, short-term mortality, and quality of life [11]. However, a meta-analysis result from 5,264 HF patients showed that self-management interventions were ineffective in reducing readmission rate [12]. Some studies have suggested that the low efficacy of self-management interventions was caused by the knowledge gap between health care professionals (HCPs) and patients [13, 14]. A previous study reported that fewer than 10 % of all patients who received discharge education understood what they had learned [15]. In addition, HF patients adhered to their prescribed medication regimes but did not follow the recommended behavioral changes, including physical activity and weight monitoring [16]. These findings indicate that effective discharge education is needed to improve self-management among HF patients.

Educational interventions using the teach-back method (TBM) for chronically ill patients who have difficulties in self-management have improved knowledge, adherence, self-efficacy, self-care skills [17], and readmission rates [18]. The TBM is defined as a communication confirmation method used by HCPs to confirm whether a patient or caregiver understands what is being explained to them [19, 20]. Several studies have identified the effectiveness of TBM for discharge education, finding that it increased knowledge retention, improved self-care, and reduced the readmission rate for HF patients [21–24]. Boyde et al.’s (2018) randomized controlled trial showed that self-care educational intervention using TBM for HF patients effectively reduced unplanned hospital readmission by 30 % [21]. Also, Dihn et al.’s (2019) presented that the discharge education program using TBM for patients with HF improved knowledge and self-care maintenance [22]. These results indicated that the discharge education using TBM for HF patients could be improved not only the knowledge aspect but also the clinical outcomes. However, previous studies lacked a detailed description of the program development process.

To our knowledge, no previous study used TBM in Korea. Several studies to improve self-management among HF patients were recently conducted in Korea, but they focused only on a health diary [25] and telephone follow up [26]. They did not consider effective education strategies such as TBM or essential outcomes including readmission rate. Therefore, our aims in this study were to develop a discharge education program using TBM, to evaluate the quality of the program, and to share the program development process in detail with researchers and HCPs.

## Methods

### Study design

We used a methodological study design to develop a discharge education program with TBM for HF patients and their caregivers.

### Methodological model

We developed our program by applying the analysis, design, development, implementation, and evaluation (ADDIE) model [27]. The current study focuses on phases 1–3 (analysis, design, and development).

### Phase 1: Analysis

The analysis phase includes clarifying problems, determining goals, and confirming the intended population [27]. In this study, we conducted a needs assessment by analyzing electronic medical records (EMR), performing a systematic literature review, and conducting focus group interviews.

#### EMR data analysis

We used EMR data to retrospectively analyze the 30-day readmission rate for 295 HF patients discharged from a tertiary hospital in Korea. The unplanned readmission rate of HF patients in that hospital was 19 % [28].

#### Systematic reviews

A systematic review and meta-analysis were conducted to identify the effectiveness of discharge education using TBM. We found that discharge education with TBM reduced the 30-day readmission rate by 45 %. The detailed methods for the systematic review and meta-analysis are described in our previous work [18].

#### Focus group interviews

Focus group interviews were conducted to explore the unmet needs for discharge education among cardiovascular disease patients and their nurses. Eighteen patients and five nurses participated in the interviews. Cardiovascular disease patients reported unmet needs of discharge education in terms of medication effects/side effects, detailed information on exercise/nutrition, and applying the right information at the right time. On the other side, nurses experienced unmet needs in the absence of a patient-oriented standard manual, insufficient time, and unified learning methods (pamphlet). Our program was developed based on these results.

### Phase 2: Designing the HEART program

The design phase focuses on objectives, assessment instruments, and planning using a systematic and specific approach [27]. The program is generated and validated during the development phase [27]. In this study, we designed and developed the Heart failure care for Enhancing self-management At home by Reinforcing discharge education with Teach-back method (HEART) using Donabedian’s structure, process, and outcome model [29] of healthcare service delivery.

### Structure

We designed the structure of the program by defining the target population, providers, and when and where to deliver the program. The target population is adult patients diagnosed with HF and scheduled to be discharged to their homes. The providers are nurses who provide discharge education to patients and their caregivers in a cardiology ward. The timepoint is the scheduled date of discharge or the afternoon before discharge. The place is a private patient room or consultation room.

### Process

**Fig. 1:**
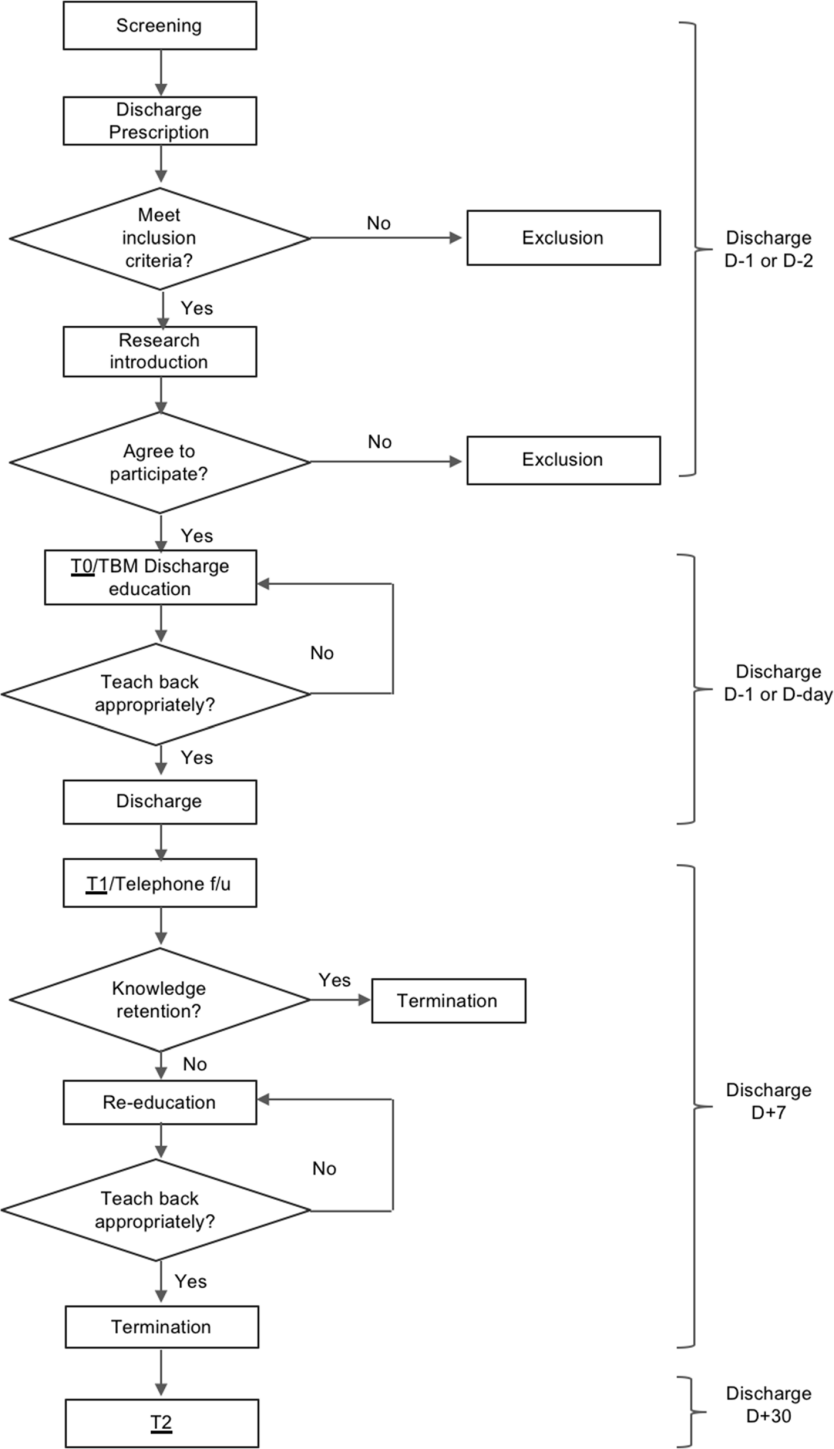
Process algorithm

The process for the HEART program is based on the hospital’s discharge protocol, as shown in Fig. 1. First, the researcher screens the patients for eligibility using EMR. Once patient discharge is determined, the researcher visits the room and explains the study. If the patient decides to participate in the study, informed consent is obtained, and the pre-test (T0) is administered. Researchers (nurses) provide discharge education using TBM to patients and caregivers in the afternoon before or on the morning of discharge. If the patient or caregiver does not understand the contents, re-education is performed until the patients or caregivers understand. One week after discharge, the first post-test (T1) is conducted via telephone follow-up. If additional education regarding self-care is required, TBM education is provided until the patients or caregivers understand. One month after discharge, telephone follow-up and the second post-test (T2) are conducted.

### Outcome

We reviewed assessment instruments to test the effects of the HEART program. Table 1 summarizes the outcome measures and their reliabilities. To evaluate the HEART program, we use self-care [30], self-efficacy [31], symptoms [32], satisfaction, dependence on caregivers, and unplanned healthcare resource utilization.

**Table 1:** Outcomes

Concept	Scale	Reliability
Self-care	SCHFI version 7.2	0.70–0.85
Self-efficacy	Self-care confidence	0.84
Symptom	SSQ-HF	0.80
Satisfaction	CSQ-8	0.93
Dependence on caregiver		-
Healthcare resource utilization		-

*SCHFI *self-care heart failure index, *SSQ-HF *symptom status questionnaire-heart failure, *CSQ *client satisfaction questionnaire

### Phase 3: Development

The development phase is to generate and validate the program [22]. We developed the program contents according to educational materials from the Korean Society of Heart Failure and the American Heart Association [33, 34].

We evaluated the content validity of the program using the content validity index (CVI) [35]. The item (I)-CVI and overall CVI were evaluated using Lynn’s (1986) criteria (1 = not relevant, 2 = somewhat relevant, 3 = quite relevant, 4 = highly relevant) [35]. For each item, the I-CVI was computed as the number of “3” and “4” ratings (categorized as *valid*) divided by the total number of experts. The overall CVI was calculated as the proportion of items judged to be valid [35]. The expert panel consisted of 6 nursing professors, 4 cardiologists, 3 nurse practitioners, 3 unit managers, and 1 clinical dietician. An email questionnaire was sent to each expert, and 2 cardiologists did not respond (response rate: 88.2 %).

We also evaluated the appropriateness of the education material using the Patient Education Materials Assessment Tool for Printable Materials (PEMAT-P), which contains items to evaluate understandability and actionability for users [36]. The 17 understandability items and 7 actionability items were evaluated as “agree” (1 point), “disagree” (0 point), and “not applicable” by the participants. The percentage of agreement among participants was calculated as the understandability and actionability of the program overall. The PEMAT-P questionnaires were completed by 10 nurses who care for HF patients in cardiology wards and 10 patients with HF. There were no nonresponders.

### Ethical considerations

This study was approved by our institutional review board (No. 4-2018-0788). All methods were carried out in accordance with relevant guidelines and regulations. We also received the agreement of cardiologists and the cardiology unit to conduct the study. All the participants were given information about the purpose, procedures, and possible benefits and risks of the study. Then, they were given time to consider their participation and took part in the study voluntarily.

## Results

### HEART program

The HEART program contains seven topics for HF patients after discharge: definition of HF, medication, symptom management, weight management, dietary management, physical activity, and other precautions. The specific contents are summarized in Table 2. We developed the HEART program using the teach-back module of the Agency for Healthcare Research and Quality (AHRQ) [37], which was translated into Korean with official permission from the AHRQ. The educational material for patients was produced as a printed booklet, and the teach-back education module for HCPs was produced as slides.

**Table 2:** Overview of the HEART program

Topics	Contents	
1. Definition	- What is heart failure - What are the causes of heart failure?	
2. Medication	- Medications to take after discharge - Tips for not forgetting to take medication	
3. Symptom management	- Importance of symptom monitoring - How to manage symptoms - Critical symptoms requiring HCPs	
4. Weight management	- Importance of weight management - Tips for not forgetting weight measurements - Significant changes in weight to tell HCPs	
5. Dietary management	- Importance of dietary management - Foods to be restricted - Tips for cooking healthy food	
6. Physical activity	- Importance of physical activity - Exercise methods for heart failure patients - Tips for regular exercise	
7. Other precautions	- No smoking or drinking - Stress management - Vaccination - Regular medical examinations	
Educational materials		
	
- For patients and caregivers - Booklet		- For HCPs - Slides

*HCPs *health care professionals

The knowledge gap between a nurse and a patient or caregiver is assessed using pre-structured teach-back questions. We developed 15 questions that correspond to knowledge, attitude, and skills for each of the five major educational topics: medication, symptom management, weight management, dietary management, and physical activity (Table 3).

**Table 3:** Teach-back questions

Category	Knowledge	Attitude	Skill
Medication	What is the name of your water pill?	Why is it important to take a diuretic at a fixed time every day?	What will you do to make sure you do not forget to take your diuretics every day?
Weight management	How many kilograms per week do you need to change to call your HCPs?	Why is it important to weigh at the same time every day?	What will you do to remember to monitor your weight every day?
Dietary management	What are some foods you should avoid?	Why is it important to eat fewer salty foods?	What will you do to reduce your salty food intake?
Symptom management	What symptoms should provoke you to contact the hospital?	Why is it important to watch for heart failure symptoms every day?	What will you do to check for heart failure symptoms every day?
Physical activity	What kind of exercise are you going to do?	Why is it important to exercise regularly?	What will you do to exercise regularly?

*HCPs *health care professionals

### The validity of the HEART program

The overall CVI score for the contents of the HEART program was 0.96. The range of the item CVIs was 0.80 to 1.00. The validity of the educational content was confirmed because the CVI for all content items and for overall content were greater than 0.8.

**Fig. 2:**
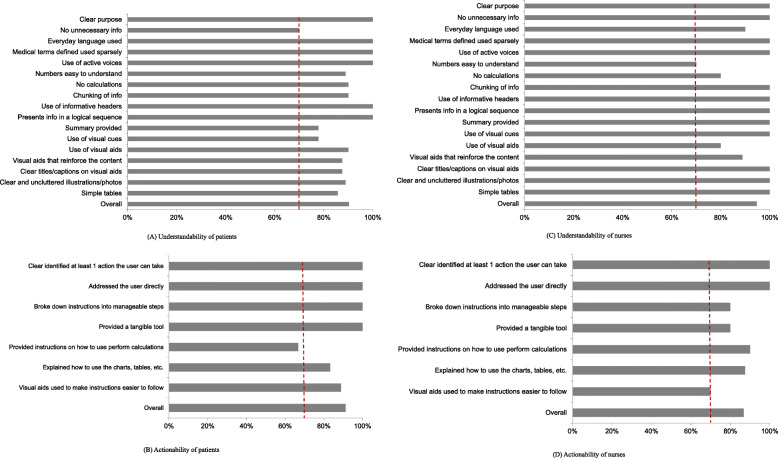
Mean understandability and actionability scores on PEMAT-P. **A** Understandability of patients. **B** Actionability of patients. **C** Understandability of nurses. **D** Actionability of nurses. *Note*. The dotted lines indicate the cut-off point (70 %) for PEMAT-P

The understandability of the educational material among patients was 90.2 %, and the actionability was 91.3 %. The understandability and actionability of the educational material among nurses were 94.6 and 86.8 %, respectively. All but one of the scores evaluating the quality of the program exceeded 70 %, the cutoff point. The patients’ response to the actionability item “Provided instruction on how to perform calculations” was 66.7 % (Fig. 2). We therefore added example data to help patients easily calculate dietary amounts.

## Discussion

Our main goal was to develop a discharge education program using TBM for HF patients. Our evaluation of the HEART program found that the content was valid, and the educational material was appropriate for both providers and patients.

The TBM was designed to deliver a discharge education program. Growing evidence indicates that discharge education using TBM for HF patients effectively improves health outcomes compared with other educational methods [22–24]. Howie-Esquivel and colleagues (2015) compared 548 elderly patients who received TBM education with 485 elderly patients who received usual care. The 30-day readmission rate in the TBM group was significantly lower than in the control group (12 % TBM group vs. 19 % usual care group). Recently, Dihn et al. (2019) conducted a cluster randomized controlled trial to test the effectiveness of a discharge education program using TBM among 140 adult patients with HF in Vietnam [22]. They found that the TBM group had significantly higher knowledge and self-care maintenance than the control group. However, those previous studies lacked detailed information about how TBM was used in the education programs. We are contributing methodologically by describing and sharing our program development process in detail. We have also added teach-back questions about attitude and skill, in addition to teach-back questions about self-management knowledge. Of course, TBM is a way to identify and close the knowledge gap between HCPs and patients [19, 20]; therefore, measuring knowledge is the most important metric. However, given that the ultimate goal of educational interventions to improve self-management is to change behaviors and attitudes [11], teach-back questions about attitudes and skills could also be helpful.

We used the ADDIE model to develop our discharge education program. ADDIE is a well-known instructional-systems design model that education technologists and instructional designers use for curriculum development [38]. The strengths of ADDIE include a systematic approach to generating a program and a robust and logical process for developing a program. It also provides an essential process to design engaging learning and training programs [38]. However, it does not address the needs and factors during the analysis process. We overcame the shortcomings of the ADDIE model by conducting a needs assessment using focus group interviews during the analysis phase. We recommend performing a needs assessment when using the ADDIE model to develop training programs for patients.

The content validity and user validity of the HEART program were deemed suitable by patients and providers. In particular, patients rated the understandability as 90.2 % and the actionability as 91.3 %. Most previous studies did not report results from evaluation of the suitability of their patient educational materials [22–24] and thus cannot be compared directly with our findings. However, this is a fairly high score compared with patient evaluations of HF information available through websites [39]. A recent analysis of 46 websites found that the overall mean understandability was 56.3 %, and the overall mean actionability was 34.7 % [39]. The PEMAT has excellent measurement properties and is useful in evaluating the quality of educational materials [36]. Therefore, future researchers should consider evaluating their educational materials from a patient perspective.

There are several limitations that we should consider for the next steps. First, this program was developed for only one tertiary hospital in Korea, which means restriction of the generalizability. To test external validity, more discharge educational program using TBM should be implemented and evaluated as the experimental studies or the quality improvement project at various settings. Second, the outcome measurement for self-care was accounted for on the self-reported questionnaire, so we cannot assume the real change in behaviors. To overcome this limitation, monitoring objective health behaviors using internet of medical things devices could be one way.

## Conclusions

The contents of the HEART program were valid, and the educational material was appropriate for both patients and nurses. We expect our structured discharge education program using TBM to enhance the self-management of HF patients. Our study shows how HCPs and researchers can practically develop an intervention program using a methodological model and systematic approach. The detailed phases by which we developed the HEART program illustrate essential elements that HCPs need to consider during implementation. Also, the structure, process, and results of the program designed in this study could guide both research and practice and can be used in settings other than Korea. Finally, the teach-back questions we use to identify gaps between HCPs and HF patients in knowledge, skill, and attitude regarding self-management could be used in clinical practice.

## Data Availability

The datasets generated and/or analysed during the current study are available from the corresponding author on reasonable request.

## Abbreviations

HF: Heart failure
TBM: Teach-back method
ADDIE: Analysis, design, development, implementation, and evaluation
CVI: Content validity index
HCPs: Health care professionals
HRRP: Hospital readmission reduction program
AHRQ: Agency for Healthcare Research and Quality
HEART: Heart failure care for Enhancing self-management At home by Reinforcing discharge education with Teach-back method
PEMAT-P: Patient education materials assessment tool for printable materials

## Declarations

**Ethical approval and consent to participate**.

This study was approved by the institutional review board at the Severance Hospital, Yonsei University Health System (4-2018-0788). A written consent form was obtained from each participant voluntarily before data collection.

## References

[1] American Heart Association (AHA). What is heart failure? 2017. https://www.heart.org/en/health-topics/heart-failure/what-is-heart-failure. Accessed 25 Nov 2019.

[2] Ambrosy AP, Fonarow GC, Butler J, Chioncel O, Greene SJ, Vaduganathan M, Nodari S, Lam CSP, Sato N, Shah AN (2014). The global health and economic burden of hospitalizations for heart failure: lessons learned from hospitalized heart failure registries. J Am Coll Cardiol.

[3] Centers for Medicare and Medicaid Services (CMS). Hospital Readmissions Reduction Program (HRRP). 2012. https://www.cms.gov/Medicare/Medicare-Fee-for-Service-Payment/AcuteInpatientPPS/Readmissions-Reduction-Program.html. Accessed 15 Oct 2019.

[4] Cowie MR, Anker SD, Cleland JGF, Felker GM, Filippatos G, Jaarsma T, Jourdain P, Knight E, Massie B, Ponikowski P (2014). Improving care for patients with acute heart failure: before, during and after hospitalization. ESC Heart Fail.

[5] Lee JH, Lim NK, Cho MC, Park HY (2016). Epidemiology of heart failure in Korea: present and future. Korean Circ J.

[6] Chung JE, Noh E, Gwak HS (2017). Evaluation of the predictors of readmission in Korean patients with heart failure. J Clin Pharm Ther.

[7] Gupta A, Allen LA, Bhatt DL, Cox M, DeVore AD, Heidenreich PA, Hernandez AF, Peterson ED, Matsouaka RA, Yancy CW (2018). Association of the hospital readmissions reduction program implementation with readmission and mortality outcomes in heart failure. JAMA Cardiol.

[8] Díez-Villanueva P, Alfonso F (2016). Heart failure in the elderly. J Geriatr Cardiol.

[9] Toukhsati SR, Driscoll A, Hare DL (2015). Patient self-management in chronic reart failure - establishing concordance between guidelines and practice. Card Fail Rev.

[10] Horwitz L and Krumholz H. Heart failure self-management. 2018. https://www.uptodate.com/contents/heart-failure-self-management. Accessed 26 Nov 2019.

[11] Riegel B, Dickson VV, Faulkner KM (2016). The situation-specific theory of heart failure self-care: revised and updated. J Cardiovasc Nurs.

[12] Jonkman NH, Westland H, Groenwold RH, Agren S, Atienza F, Blue L, de la Bruggink-AndrePorte PW, DeWalt DA, Hebert PL, Heisler M (2016). Do self-management interventions work in patients with heart failure? An individual patient data meta-analysis. Circulation.

[13] Hansen LO, Greenwald JL, Budnitz T, Howell E, Halasyamani L, Maynard G, Vidyarthi A, Coleman EA, Williams MV (2013). Project BOOST: effectiveness of a multihospital effort to reduce rehospitalization. J Hosp Med.

[14] Jack BW, Chetty VK, Anthony D, Greenwald JL, Sanchez GM, Johnson AE, Forsythe SR, O’Donnell JK, Paasche-Orlow MK, Manasseh C (2009). A reengineered hospital discharge program to decrease rehospitalization: a randomized trial. Ann Intern Med.

[15] Regalbuto R, Maurer MS, Chapel D, Mendez J, Shaffer JA (2014). Joint Commission requirements for discharge instructions in patients with heart failure: is understanding important for preventing readmissions?. J Card Fail.

[16] Jaarsma T, Stromberg A, Ben Gal T, Cameron J, Driscoll A, Duengen HD, Inkrot S, Huang TY, Huyen NN, Kato N (2013). Comparison of self-care behaviors of heart failure patients in 15 countries worldwide. Patient Educ Couns.

[17] Ha Dinh TT, Bonner A, Clark R, Ramsbotham J, Hines S (2016). The effectiveness of the teach-back method on adherence and self-management in health education for people with chronic disease: a systematic review. JBI Database System Rev Implement Rep.

[18] Oh EG, Lee HJ, Yang YL, Kim YM. Effectiveness of dischar ge education with the Teach-Back Method on 30-day readmission: a systematic review. J Patient Saf. 2021;17(4):305–10. 10.1097/PTS.0000000000000596 30882616

[19] National Library of Medicine (NLM). Teach-back communication. 2014. https://www.ncbi.nlm.nih.gov/mesh/?term=teach-back+communication. Accessed 10 Oct 2019.

[20] Wikipedia. Teach-back method. 2017. https://en.wikipedia.org/wiki/Teach-back_method. Accessed 10 Oct 2019.

[21] Boyde M, Peters R, New N, Hwang R, Ha T, Korczyk D (2018). Self-care educational intervention to reduce hospitalisations in heart failure: a randomised controlled trial. Eur J Cardiovasc Nurs.

[22] Dinh HTT, Bonner A, Ramsbotham J, Clark R (2019). Cluster randomized controlled trial testing the effectiveness of a self-management intervention using the teach-back method for people with heart failure. Nurs Health Sci.

[23] Howie-Esquivel J, Carroll M, Brinker E, Kao H, Pantilat S, Rago K, De Marco T (2015). A strategy to reduce heart failure readmissions and inpatient costs. Cardiol Res.

[24] White M, Garbez R, Carroll M, Brinker E, Howie-Esquivel J (2013). Is “teach-back” associated with knowledge retention and hospital readmission in hospitalized heart failure patients?. J Cardiovasc Nurs.

[25] Shim JL, Hwang SY (2016). Development and effects of a heart health diary for self-care enhancement of patients with weart failure. J Korean Acad Nurs.

[26] Moon MK, Yim J, Jeon MY (2018). The effect of a telephone-based self-management program led by nurses on self-care behavior, biological index for cardiac function, and depression in ambulatory heart failure patients. Asian Nurs Res.

[27] Branch RM. Instructional design: the ADDIE approach, vol. 722. New York: Springer Science & Business Media; 2009.

[28] Kim YM, Lee T, Lee HJ, Yang YL, Oh EG. Readmission of high-risk discharged patients at a tertiary hospital in Korea. J Healthc Qual. 2019;41(4):e30–7. 10.1097/JHQ.0000000000000151 30362997

[29] Donabedian A. The quality of care. How can it be assessed? JAMA. 1988;260(12):1743–1748. 10.1001/jama.260.12.1743 3045356

[30] Riegel B, Barbaranelli C, Carlson B, Sethares KA, Daus M, Moser DK, Miller J, Osokpo OH, Lee S, Brown S (2019). Psychometric testing of the revised self-care of heart failure index. J Cardiovasc Nurs.

[31] Riegel B, Carlson B, Moser DK, Sebern M, Hicks FD, Roland V (2004). Psychometric testing of the self-care of heart failure index. J Card Fail.

[32] Heo S, An M, Kim J (2017). Validation of the symptom status questionnaire-heart failure in Korean patients. Appl Nurs Res.

[33] American Heart Association (AHA). Get with the guideline – Heart failure. 2018. https://www.heart.org/en/professional/quality-improvement/get-with-the-guidelines/get-with-the-guidelines-heart-failure. Accessed 25 Nov 2019.

[34] Korean Society of Heart Failure (KSHF). Learn about heart failure. 2018. http://khfs.or.kr/know/. Accessed 15 Oct 2019.

[35] Lynn MR (1986). Determination and quantification of content validity. Nurs Res.

[36] Shoemaker SJ, Wolf MS, Brach C (2014). Development of the Patient Education Materials Assessment Tool (PEMAT): a new measure of understandability and actionability for print and audiovisual patient information. Patient Educ Couns.

[37] Agency for Healthcare Research and Quality (AHRQ). Teach-back: interactive module slides. 2017. https://www.ahrq.gov/professionals/quality-patient-safety/patient-family-engagement/pfeprimarycare/interventions/teachback-module.html. Accessed 15 Oct 2018.

[38] Morrison GR, Ross SJ, Morrison JR, Kalman HK. Designing effective instruction. Hoboken: Wiley; 2019.

[39] Cajita MI, Rodney T, Xu J, Hladek M, Han H-R (2017). Quality and health literacy demand of online heart failure information. J Cardiovasc Nurs.

